# SARS‐CoV‐2 Omicron emergence urges for reinforced One‐Health surveillance

**DOI:** 10.15252/emmm.202115558

**Published:** 2022-01-27

**Authors:** Xavier Montagutelli, Sylvie van der Werf, Felix A Rey, Etienne Simon‐Loriere

**Affiliations:** ^1^ Mouse Genetics Laboratory Institut Pasteur, Université de Paris Paris France; ^2^ Molecular Genetics of RNA Viruses Unit CNRS UMR 3569 Institut Pasteur, Université de Paris Paris France; ^3^ National Reference Center for Respiratory Viruses Institut Pasteur Université de Paris Paris France; ^4^ Structural Virology Unit CNRS UMR 3569 Institut Pasteur, Université de Paris Paris France; ^5^ G5 Evolutionary Genomics of RNA Viruses Institut Pasteur, Université de Paris Paris France

**Keywords:** Evolution & Ecology, Microbiology, Virology & Host Pathogen Interaction

## Abstract

SARS‐CoV‐2 Omicron harbors substitutions in the receptor binding domain of the spike which strongly suggest its capacity to infect rodents. Wild animal reservoirs could favor the emergence of new variants with risks of spillback to humans and should be closely monitored.

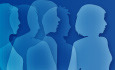

Throughout its short history, SARS‐CoV‐2 has demonstrated its capacity to jump back and forth between humans and animals. Following its emergence from a likely bat reservoir, with or without an intermediate host, the virus has successfully infected an array of domestic and wild animals (Chandler *et al*, 2021; OIE, 2022). In 2020, the infection of mink farms in multiple countries was a strong reminder of the importance of One‐Health surveillance. The constitution of a non‐human reservoir from where the virus could spill back into humans is a major concern, as exemplified by multiple viruses, including influenza, with documented cases of human‐to‐swine‐to‐human transmission (Glud *et al*, 2021). Importantly, the intensive circulation of an emerging virus in a new host leads to the accumulation of amino acid changes, some reflecting adaptation to this novel environment, which could result in the evolution of variants with unpredictable properties with regard to human infection. As a case in point, SARS‐CoV‐2 spillback from minks to humans in Denmark was associated with lower capability of pre‐existing human antibodies to neutralize the variant (Larsen *et al*, 2021).

Living close to humans, sometimes in large numbers, rodents are susceptible to infectious diseases that can threaten human health, such as plague, leptospirosis, or Lassa fever. In March 2021, we reported that mice, which are not susceptible to the initial SARS‐CoV‐2 genotype, were permissive to viral replication of the Alpha, Beta, and Gamma Variants of Concern (VOC; preprint: Montagutelli *et al*, 2021b), a finding that was later confirmed by others. We also demonstrated that Beta could be transmitted between mice by direct contact (preprint: Montagutelli *et al*, 2021b). Central to this host range expansion is the N501Y substitution in the receptor‐binding domain of the spike, a convergent change detected in many SARS‐CoV‐2 lineages.

The newly emerged Omicron VOC harbors not only N501Y but also changes at residues 493 and 498 to basic amino acids as seen recurrently in mouse‐adapted strains (Fig 1), arguing for its capacity to replicate in mice. The intense circulation of variants presenting these features is a stark warning of the risk of reservoirs of SARS‐CoV‐2 in wild rodents, and this risk could extend to other animal species with sufficient population densities. In fact, the genomic variations of Omicron are so extensive that they could have been acquired upon circulation in an animal reservoir, a credible alternative hypothesis for its emergence process.

**Figure 1:**
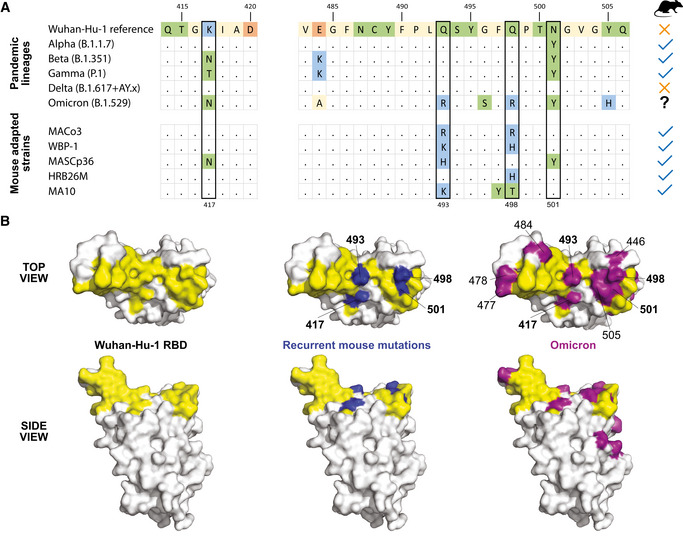
Amino acid changes in the receptor–binding domain (RBD) of the spike of SARS‐CoV‐2 Residues 417, 493, 498, and 501 are also modified in several mouse‐adapted SARS‐CoV‐2 strains. A: Table of changes in section of the RBD for pandemic lineages and mouse‐adapted strains. B: Mapping onto the X‐ray structure of the RBD (PDB 6M0J) shown as a white surface with the human ACE2 contact area highlighted in yellow (left panels). The RBD residues recurrently observed as undergoing changes upon adaptation of SARS‐CoV‐2 to mice are marked in blue in the middle panels, while the multiple changes observed in the RBD of the Omicron variant spike are indicated in purple in the right panels. Mouse‐adapted strains: MACo3 from Montagutelli *et al* (preprint: Montagutelli *et al*, 2021a), WBP‐1 from Huang *et al* (2021), MASCp36 from Sun *et al* (2021), HRB26M, which also contains a deletion near the furin site, from Wang *et al* (2020) and MA10 from Leist *et al* (2020). Other changes outside of the RBD were also noted in mouse‐adapted SARS‐CoV‐2 genomes.

We thus call for practical reinforcements of collaborative efforts of surveillance at the human–animal interface and its environment—both in high‐density cities and rural places for rodents—as a critical determinant of current and future pandemic risk.

## Author contributions

XM and ES‐L designed and coordinated the study. FAR performed structural analysis. XM and ES‐L wrote and revised the manuscript with input from SW and FAR.

## Supporting information


